# Unorthodox features in two venerid bivalves with doubly uniparental inheritance of mitochondria

**DOI:** 10.1038/s41598-020-57975-y

**Published:** 2020-01-23

**Authors:** Charlotte Capt, Karim Bouvet, Davide Guerra, Brent M. Robicheau, Donald T. Stewart, Eric Pante, Sophie Breton

**Affiliations:** 1 Department of Biological Sciences, Université de Montréal, Montréal, QC Canada; 2 Department of Biology, Dalhousie University, Halifax, NS Canada; 3 Department of Biology, Acadia University, Wolfville, NS B4P 2R6 Canada; 4 Littoral, Environnement et Sociétés (LIENSs), UMR 7266 CNRS–La Rochelle Université, 2 rue Olympe de Gouges, 17000 La Rochelle, France

**Keywords:** Evolution, Genetics, Zoology

## Abstract

In animals, strictly maternal inheritance (SMI) of mitochondria is the rule, but one exception (doubly uniparental inheritance or DUI), marked by the transmission of sex-specific mitogenomes, has been reported in bivalves. Associated with DUI is a frequent modification of the mitochondrial *cox2* gene, as well as additional sex-specific mitochondrial genes not involved in oxidative phosphorylation. With the exception of freshwater mussels (for 3 families of the order Unionida), these DUI-associated features have only been shown in few species [within Mytilidae (order Mytilida) and Veneridae (order Venerida)] because of the few complete sex-specific mitogenomes published for these orders. Here, we present the complete sex-specific mtDNAs of two recently-discovered DUI species in two families of the order Venerida, *Scrobicularia plana* (Semelidae) and *Limecola balthica* (Tellinidae). These species display the largest differences in genome size between sex-specific mitotypes in DUI species (>10 kb), as well as the highest mtDNA divergences (sometimes reaching >50%). An important in-frame insertion (>3.5 kb) in the male *cox2* gene is partly responsible for the differences in genome size. The *S. plana cox2* gene is the largest reported so far in the Kingdom Animalia. The mitogenomes may be carrying sex-specific genes, indicating that general mitochondrial features are shared among DUI species.

## Introduction

Animal mitochondrial DNA (mtDNA) is typically depicted as a strictly maternally inherited (SMI) circular DNA molecule that is relatively small (~16 kb) and genomically streamlined with almost invariant gene content (13 protein-coding genes and 24 structural RNAs)^1,2^. However, important deviations do occur in the mtDNAs of bivalve molluscs, which not only display dramatic variation in size (<14.7 kb to >67 kb)^3,4^ and gene arrangement^5^, but also the presence of additional protein-coding genes not associated with oxidative phosphorylation^6–9^. An even more extreme departure from the norm in bivalve mitochondrial genomes is their mode of doubly uniparental inheritance (DUI) — both egg and sperm mitochondria are transmitted from generation to generation in several bivalve species, but only male offspring retain paternally-transmitted mitochondria (with male or M mtDNA) in their gametes^10–12^. Adult females of DUI-exhibiting species usually possess only the female-transmitted mtDNA (F mtDNA) in their soma and gametes whereas males possess F mtDNA in their soma and M mtDNA in their gametes^10–13^. The DNA divergence between F and M mtDNAs usually vary from about 8% to 40% depending on the species^12,14^. Genetic analyses suggested that both F and M mtDNAs in DUI bivalves evolve at a faster rate than typical metazoan mtDNA, and that M mtDNA evolves faster than F mtDNA^15–17^. One factor explaining this observation may be that the M genome is subject to weaker selective pressures than the F genome due to an unequal “division of labor” in the DUI system^16^. Typical animal mtDNA functions in gonads and somatic tissues of both sexes whereas under DUI, F mtDNA functions in female gonads and the soma of both sexes, while M mtDNAs functions primarily in spermatozoa of male gonads and only partially in the male soma^13,16,18^. As opposed to SMI that promotes homoplasmy, a state in which all mtDNA copies are typically genetically identical in each cell, thus preventing potentially harmful genomic conflicts, DUI is a naturally heteroplasmic system in which two highly divergent mitochondrial lineages coexist in the same nuclear background, enabling the analysis of the consequences of tissue heteroplamy^13^.

In addition to a different mode of mitochondrial transmission and evolution rate of mtDNA, two other remarkable differences have been reported between the F and M mtDNAs in DUI bivalves. First, the COX2 protein encoded by M mtDNA (M*cox2* gene) is longer than the FCOX2 protein, which is about the same size as other animal COX2 proteins, although this pattern is not shared by all DUI species for which sex-specific mtDNAs have been completely sequenced (reviewed in Bettinazzi *et al*.^19^). For example, male freshwater mussels (order Unionida) have an approximately 550 bp 3′-coding extension to the *cox2* gene (M*cox2*e), that is absent from other animals mtDNAs^20–22^. This extension proved to be translated and localized in both inner and outer mitochondrial membranes^23,24^, leading to the hypothesis that it could act as a mitochondrial tag implicated in paternal mitochondria survival in male embryos^21^. Such a 3′-coding extension of the M*cox2* gene has also been found in the mytilid mussel *Musculista senhousia*, but in a duplicated version of the *cox2* gene^25^. The extension is apparently absent from M mtDNAs of other mytilid DUI species (e.g., *Mytilus* spp.)^12,26^. In the family Veneridae, the M mtDNA of *Meretrix lamarckii* presents an insertion of 100 codons within the *cox2* gene^19^, whereas a duplicated version of the *cox2* gene, similar to that of *M. senhousia* (i.e., longer at 3′), has been found in *Ruditapes philippinarum*, but located in the F genome (unpublished GenBank annotation mentioned in Passamonti *et al*.^25^). As suggested by Bettinazzi *et al*.^19^, non-canonical features of the *cox2* gene are often coupled with DUI, but it is difficult to propose a general function because each major lineage of bivalves that possesses the DUI system exhibits some novel features. Clearly further analyses involving additional species are required to better understand the relationship between these structural variations in the *cox2* gene and DUI, as well as other general features of DUI-exhibiting mitochondrial genomes.

To date, DUI has been found in over one hundred bivalve species representing four taxonomic orders and twelve bivalve families (reviewed in Gusman *et al*.^27^). In these species, complete F and M mtDNAs have been sequenced for ∼25 DUI spp. of the families Mytilidae (order Mytilida), Veneridae (order Venerida), Unionidae, Margaritiferidae and Hyriidae (order Unionida)^6,7,22,28–35^. To our knowledge, all these species share one DUI-specific feature: they contain additional sex-specific mitochondrial genes without recognizable homologies to other known genes (hereafter called mitochondrial ORFans or mtORFans). These F- and M-specific mtORFans (which have been shown to be expressed) have been respectively called F-*orf* and M-*orf* ^6–8,36–38^. This discovery is particularly interesting because these mtORFans could be responsible for the different mode of transmission of the mtDNAs and/or the functioning of DUI in bivalves. In unionid freshwater mussels, their discovery established a strong link between DUI and the maintenance of gonochorism (hermaphroditic species possess SMI and usually a highly modified F-*orf* gene, called H-*orf*)^7,22,39^. Otherwise, their predicted functions support their direct involvement in the DUI mechanism: F-*ORF* proteins are suggested to interact with nucleic acids, adhere to membranes, and have roles in signalling, and M-*ORF*s are suggested to interact with the cytoskeleton and take part in ubiquitination and apoptosis^7,8,40^. However, the precise nature of the link between DUI and sex determination and the functions of the F and M mtORFans remains currently unknown. Again, further analyses involving additional species are needed to shed light on this.

Recently, DUI has been discovered in *Scrobicularia plana*^27^, a species of the family Semelidae (order Venerida)^41^, as well as in *Limecola balthica*^42^, a species of the family Tellinidae (order Venerida)^41^, and except for the complete F mtDNA of *L. balthica*^43^, their M mtDNAs and F mtDNA, for *S. plana*, have not previously been sequenced or reported on until now. In this study, we present the complete M and F mitochondrial genomes of these two DUI species. Our main objective was to highlight both unique features and characteristics shared among DUI species of different bivalve families. Obtaining the complete sex-specific mitogenome sequences of the species *S. plana* was particularly interesting to better understand the hypothesized link between DUI and sex determination since an “intersex” condition, i.e., the appearance of oocytes in male gonads following endocrine disruption, has been reported in this species and was associated with down-regulation of male mitochondrial transcripts in males exhibiting intersex compared to “normal” males (see Gusman *et al*.^27^ for details). Overall, besides indicating that the newly sequenced mitogenomes may be carrying sex-specific genes like in other DUI species, our data reveal that the *cox2* gene in the M mitogenome of *S. plana* is the largest reported so far in the Kingdom Animalia. It remains to be demonstrated if such unorthodox features play key roles in DUI and sex determination in bivalves.

## Results and Discussion

### Main genomic features

**Figure 1:**
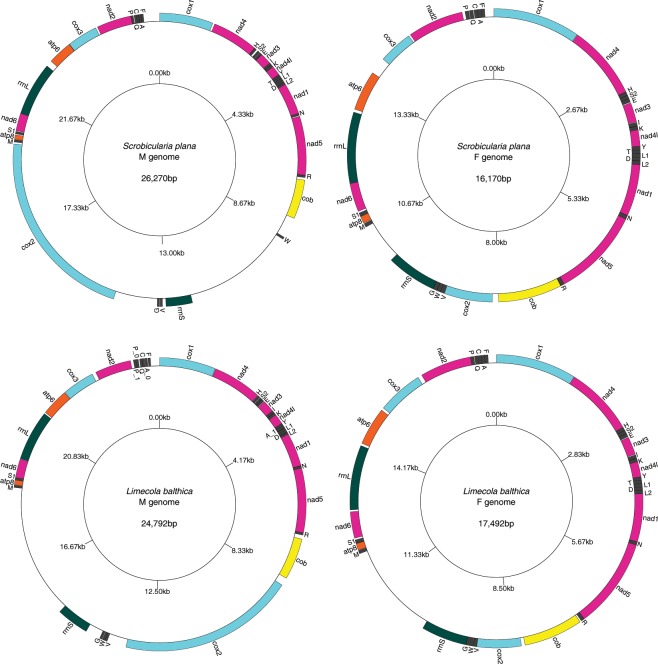
Gene maps of M and F genomes of *Scrobicularia plana* and *Limecola balthica*. All genes are encoded on the heavy strand, total genome lengths are reported inside their corresponding genome. Gene colors correspond to functional groups (OXPHOS gene families, tRNAs and rRNAs).

The complete F mtDNAs of *Scrobicularia plana* and *Limecola balthica* are 16,170 bp and 17,492 bp in length, respectively, whereas the complete M mtDNAs are respectively 26,270 bp and 24,792 bp long (Fig. 1). To our knowledge, these values represent the biggest differences in genome size between the F and M mtDNAs in species with DUI (Table 1), which usually do not exceed 2 kb. These differences in length are partly explained by the presence of an insertion in the protein-coding gene (PCG) *cox2* in the M mtDNA of both species (F*cox2* = 855 bp and M*cox2* = 4,815 bp in *L. balthica*, whereas F*cox2* = 861 bp and M*cox2* = 5,679 bp in *S. plana*) (Table 2), which is discussed in more details below. Otherwise, the two sex-specific mtDNAs in each species possess the 13 typical mitochondrial PCGs of metazoans^1^, all present on the same strand, as is the case in most bivalve species^14,19,44^. Gene organisation is similar among the four genomes, except for the region between *cytb* and *trnM* in the M mtDNA of *S. plana*. Reorganisation events, such as inversion or transposition, as well as tandem duplication followed by random loss events, are common in animals mtDNAs^1^.

**Table 1:** Mitochondrial genome sizes in DUI species exhibiting extreme length differences between M and F types.

Species	Mitotype	Genome Size (bp)	Genome size difference (bp)	GenBank Accession Number	Reference
**Freshwater mussels (Unionida, Unionidae)**					
*Anodonta anatina*	F	15,637		KF030965	28
*Anodonta anatina*	M	16,906	1,269	KF030963	29
**Freshwater mussels (Unionida, Hyriidae)**					
*Hyridella menziesi*	F	16,031		KU873121	22
*Hyridella menziesi*	M	18,140	2,109	KU873122	22
**Freshwater mussels (Unionida, Margaritiferidae)**					
*Cumberlandia monodonta*	F	16,099		KU873123	22
*Cumberlandia monodonta*	M	17,575	1,476	KU873124	22
**Marine mussels (Mytilida, Mytilidae)**					
*Musculista senhousia*	F	21,557		GU001953	17
*Musculista senhousia*	M	20,621	936	GU001954	17
**Marine clams (Venerida, Veneridae)**					
*Meretrix lamarckii*	F	20,025		KP244451	19
*Meretrix lamarckii*	M	19,688	337	KP244452	19
**Marine clams (Venerida, Tellinidae)**					
*Limecola balthica*	F	17,492		MN528028	Present study
*Limecola balthica*	M	24,792	7,300	MN528029	Present study
**Marine clams (Venerida, Semelidae)**					
*Scrobicularia plana*	F	16,170		MN528026	Present study
*Scrobicularia plana*	M	26,270	10,100	MN528027	Present study

**Table 2:** General features of *Scrobicularia plana* and *Limecola balthica* M and F mitochondrial genomes.

*Scrobicularia plana*			*Scrobicularia plana*			*Limecola balthica*			*Limecola balthica*		
F mt genome			M mt genome			F mt genome			M mt genome		
Gene/Region	Length	Start/Stop	Gene/ Region	Length	Start/Stop	Gene/ Region	Length	Start/Stop	Gene/ Region	Length	Start/Stop
*cox1*	1554	ATA/TAA	*cox1*	1551	ATT/TAA	*cox1*	1554	ATA/TAA	*cox1*	1536	ATT/TAA
*nad4*	1323	TTG/TAG	*nad4*	1335	GTG/TAA	*nad4*	1341	ATA/TAG	*nad4*	1341	TTG/TAA
*trnH*	65		*trnH*	63		*trnH*	64		*trnH*	64	
*trnS2*	64		*trnS2*	65		*trnS2*	65		*trnS2*	64	
*trnE*	66		*trnE*	64		*trnE*	67		*trnE*	66	
*nad3*	348	ATA/TAA	*nad3*	348	ATA/TAA	*nad3*	348	ATA/TAG	*nad3*	348	ATG/TAA
*trnI*	68		*trnI*	68		*trnI*	69		*trnL*	68	
*trnK*	64		*trnK*	66		*trnK*	65		*trnK*	65	
*nad4L*	291	ATT/TAA	*nad4L*	285	ATT/TAG	*nad4L*	291	GTG/TAA	*nad4L*	276	TTG/TAG
*trnY*	64		*trnY*	64		*trnY*	63		*trnY*	67	
*trnT*	63		*trnT*	64		*trnT*	64		*trnA_1*	71	
*trnL1*	66		*trnL1*	65		*trnL1*	66		*trnL1*	65	
*trnD*	63		*trnD*	66		*trnD*	63		*trnD*	65	
*trnL2*	66		*trnL2*	66		*trnL2*	66		*trnL2*	65	
*nad1*	924	GTG/TAG	*nad1*	927	ATT/TAG	*nad1*	924	ATG/TAG	*nad1*	927	ATA/TAG
*trnN*	66		*trnN*	65		*trnN*	65		*trnN*	65	
*nad5*	1635	TTG/TAA	*nad5*	1704	ATG/TAG	*nad5*	1743	TTG/T**	*nad5*	1767	ATA/TAG
*trnR*	67		*trnR*	65		*trnR*	65		*trnR*	65	
*cob*	1135	ATA/T**	*cob*	1135	ATG/T**	*cob*	1140	ATT/TAA	*cob*	1137	ATA/TAA
*cox2*	861	ATG/TAA	*trnW*	64		*cox2*	855	ATG/TAA	*cox2*	4815^**#**^	ATG/T**
*trnV*	69		*rrnS*	765		*trnV*	66		*trnV*	65	
*trnW*	66		*trnV*	66		*trnW*	66		*trnW*	67	
*trnG*	68		*trnG*	68		*trnG*	66		*trnG*	66	
*rrnS*	896		*cox2*	5679	ATG/TAG	*rrnS*	880		*rrnS*	886	
*trnM*	66		*trnM*	67		*trnM*	65		*trnM*	64	
*atp8*	129	ATT/TAG	*atp8*	129	ATG/TAA	*atp8*	129	ATT/TAA	*atp8*	130	TTG/T**
*trnS1*	71		*trnS1*	69		*trnS1*	69		*trnS1*	69	
*nad6*	510	ATA/TAA	*nad6*	510	ATT/TAG	*nad6*	510	ATA/TAA	*nad6*	513	TTG/TAG
*rrnL*	1269		*rrnL*	1556		*rrnL*	1249		*rrnL*	1379	
*atp6*	714	ATG/TAG	*atp6*	705	ATT/TAA	*atp6*	735	ATG/TAA	*atp6*	741	GTG/TAG
*cox3*	882	ATA/TAG	*cox3*	882	ATA/TAG	*cox3*	882	ATA/TAA	*cox3*	885	GTG/TAA
*nad2*	996	ATG/TAA	*nad2*	1002	ATT/TAA	*nad2*	990	ATG/TAA	*nad2*	987	ATG/TAG
*trnP*	66		*trnP*	67		*trnP*	66		*trnP_1*	64	
*trnQ*	67		*trnQ*	67		*trnQ*	66		*trnP_0*	65	
*trnC*	63		*trnC*	61		*trnC*	61		*trnQ*	66	
*trnA*	65		*trnA*	65		*trnA*	67		*trnC*	63	
*trnF*	63		*trnF*	65		*trnF*	66		*trnA_0*	66	
									*trnF*	65	

NOTE – T** stands for incomplete stop codon; ^#^The exact size of the M*cox2* gene in *Limecola balthica* is uncertain.

The initiation and termination codons for the typical 13 PCGs encoded by the four mitogenomes are presented in Table 2. There are differences between sex-specific mtDNAs within species and also between species. For example, in *S. plana* a similar start codon between the F and M mtDNA for a particular gene is observed in 4 cases out of 13 (only two in *L. balthica*) whereas 6 PCGs out of 13 have a similar stop codon (4 in *L. balthica*). Overall, most of the PCGs use the ATD start codon (where D means A, T or G) found in metazoan mtDNAs^45^: ATA occurs the most (15/52), followed by ATG (14/52) and ATT (11/52). TTG is also found in seven cases, and GTG in five, an observation that has been previously reported in molluscan species^46,47^. Most of the PCGs are terminated with the TAA (27/52) and TAG (20/52) codons. Some incomplete termination codons are also found, which are assumed to be completed by polyadenylation of their mRNAs^48^.

The usual 22 tRNAs are found in the four mitochondrial genomes, ranging from 61 to 71 bp, and most of them can be folded into the typical secondary structures (not shown). There is an exception in the M mtDNA of *L. balthica*, which is lacking *trnT*, but instead possess a second copy of *trnA*, which has been identified by MITOS in the same area, between t*rnY* and *trnL1* (Fig. S1). This second copy of *trnA* (*trnA_1*) does not possess the typical secondary structure and it is not clear if it could be functional. This same mitochondrial genome contains also another copy of *trnP* (*trnP_1*) located next to the first *trnP* (*trnP_0*), and both possess usual secondary structures (Fig. S1). Additional tRNA copies are common in bivalve mitochondrial genomes^19,49–51^. Concerning the missing *trnT*, it is possible that the *trnA_1* could be a mutated *trnT* (with a mutation in its anticodon that changed it from *trnT* to a “secondary” *trnA*). It is also possible that an unusual secondary structure makes it unidentifiable by the tRNA search programs, and this will require further analyses.

Length variations among rRNA genes range from 765 bp to 896 bp and from 1249 bp to 1556 bp for 12S (*rrnS*) and 16S (*rrnL*), respectively (Table 2). Their locations are almost identical in the four genomes; *rrnL* is found between *nad6* and *atp6* whereas *rrnS* is found between *trnG* and *trnM*, except for the M genome of *S. plana*, in which it is located between *trnW* and *trnV* (Fig. 1; Tables 2 and 3).

**Table 3:** Number of nucleotides at gene boundaries in F and M mitochondrial genomes of *Scrobicularia plana* and *Limecola balthica*.

Boundary	*S. plana* F mtDNA		Boundary		*S. plana* M mtDNA	Boundary		*L. balthica* F mtDNA	Boundary		*L. balthica* M mtDNA
*cox1*	*nd4*	10	*cox1*	*nd4*	60	*cox1*	*nd4*	0	*cox1*	*nd4*	15
*nd4*	*trnH*	2	*nd4*	*trnH*	4	*nd4*	*trnH*	6	*nd4*	*trnH*	13
*trnH*	*trnS2*	0	*trnH*	*trnS2*	55	*trnH*	*trnS2*	1	*trnH*	*trnS2*	16
*trnS2*	*trnE*	0	*trnS2*	*trnE*	15	*trnS2*	*trnE*	1	*trnS2*	*trnE*	2
*trnE*	*nd3*	15	*trnE*	*nd3*	6	*trnE*	*nd3*	15	*trnE*	*nd3*	18
*nd3*	*trnI*	10	*nd3*	*trnI*	−17	*nd3*	*trnI*	13	*nd3*	*trnI*	−17
*trnI*	*trnK*	3	*trnI*	*trnK*	1	*trnI*	*trnK*	2	*trnI*	*trnK*	2
*trnK*	*nd4L*	0	*trnK*	*nd4L*	2	*trnK*	*nd4L*	0	*trnK*	*nd4L*	10
*nd4L*	*trnY*	1	*nd4L*	*trnY*	7	*nd4L*	*trnY*	4	*nd4L*	*trnY*	1
*trnY*	*trnT*	−3	*trnY*	*trnT*	−2	*trnY*	*trnT*	−2	*trnY*	*trnA_1*	−2
*trnT*	*trnL1*	0	*trnT*	*trnL1*	1	*trnT*	*trnL1*	0	*trnA_1*	*trnL1*	3
*trnL1*	*trnD*	−1	*trnL1*	*trnD*	−1	*trnL1*	*trnD*	−1	*trnL1*	*trnD*	−1
*trnD*	*trnL2*	2	*trnD*	*trnL2*	2	*trnD*	*trnL2*	1	*trnD*	*trnL2*	6
*trnL2*	*nd1*	1	*trnL2*	*nd1*	1	*trnL2*	*nd1*	1	*trnL2*	*nd1*	−14
*nd1*	*trnN*	3	*nd1*	*trnN*	−19	*nd1*	*trnN*	1	*nd1*	*trnN*	5
*trnN*	*nd5*	0	*trnN*	*nd5*	25	*trnN*	*nd5*	0	*trnN*	*nd5*	0
*nd5*	*trnR*	−40	*nd5*	*trnR*	4	*nd5*	*trnR*	1	*nd5*	*trnR*	−10
*trnR*	*cob*	3	*trnR*	*cob*	72	*trnR*	*cob*	31	*trnR*	*cob*	111
*cob*	*cox2*	110	*cob*	*trnW*	610	*cob*	*cox2*	71	*cob*	*cox2*	216
*cox2*	*trnV*	5	*trnW*	*rrnS*	3231	*cox2*	*trnV*	3	*cox2*	*trnV*	533
*trnV*	*trnW*	0	*rrnS*	*trnV*	101	*trnV*	*trnW*	−1	*trnV*	*trnW*	0
*trnW*	*trnG*	2	*trnV*	*trnG*	18	*trnW*	*trnG*	5	*trnW*	*trnG*	15
*trnG*	*rrnS*	4	*trnG*	*cox2*	1272	*trnG*	*rrnS*	3	*trnG*	*rrnS*	410
*rrnS*	*trnM*	732	*cox2*	*trnM*	57	*rrnS*	*trnM*	1890	*rrnS*	*trnM*	3697
*trnM*	*atp8*	11	*trnM*	*atp8*	13	*trnM*	*atp8*	9	*trnM*	*atp8*	11
*atp8*	*trnS1*	−2	*atp8*	*trnS1*	28	*atp8*	*trnS1*	3	*atp8*	*trnS1*	−1
*trnS1*	*nd6*	32	*trnS1*	*nd6*	20	*trnS1*	*nd6*	34	*trnS1*	*nd6*	−2
*nd6*	*rrnL*	0	*nd6*	*rrnL*	0	*nd6*	*rrnL*	35	*nd6*	*rrnL*	12
*rrnL*	*atp6*	54	*rrnL*	*atp6*	180	*rrnL*	*atp6*	47	*rrnL*	*atp6*	40
*atp6*	*cox3*	30	*atp6*	*cox3*	8	*atp6*	*cox3*	52	*atp6*	*cox3*	17
*cox3*	*nd2*	34	*cox3*	*nd2*	56	*cox3*	*nd2*	81	*cox3*	*nd2*	105
*nd2*	*trnP*	52	*nd2*	*trnP*	3	*nd2*	*trnP*	0	*nd2*	*trnP_1*	78
*trnP*	*trnQ*	11	*trnP*	*trnQ*	25	*trnP*	*trnQ*	13	*trnP_1*	*trnP_0*	7
*trnQ*	*trnC*	15	*trnQ*	*trnC*	0				*trnP_0*	*trnQ*	35
*trnC*	*trnA*	0	*trnC*	*trnA*	4	*trnQ*	*trnC*	−1	*trnQ*	*trnC*	0
*trnA*	*trnF*	−1	*trnA*	*trnF*	3	*trnC*	*trnA*	1	*trnC*	*trnA_0*	13
*trnF*	*cox1*	161	*trnF*	*cox1*	372	*trnA*	*trnF*	−1	*trnA_0*	*trnF*	20
						*trnF*	*cox1*	163	*trnF*	*cox1*	250

NOTE – Negative values indicate that genes overlap.

### Intraspecific divergences

**Figure 2:**
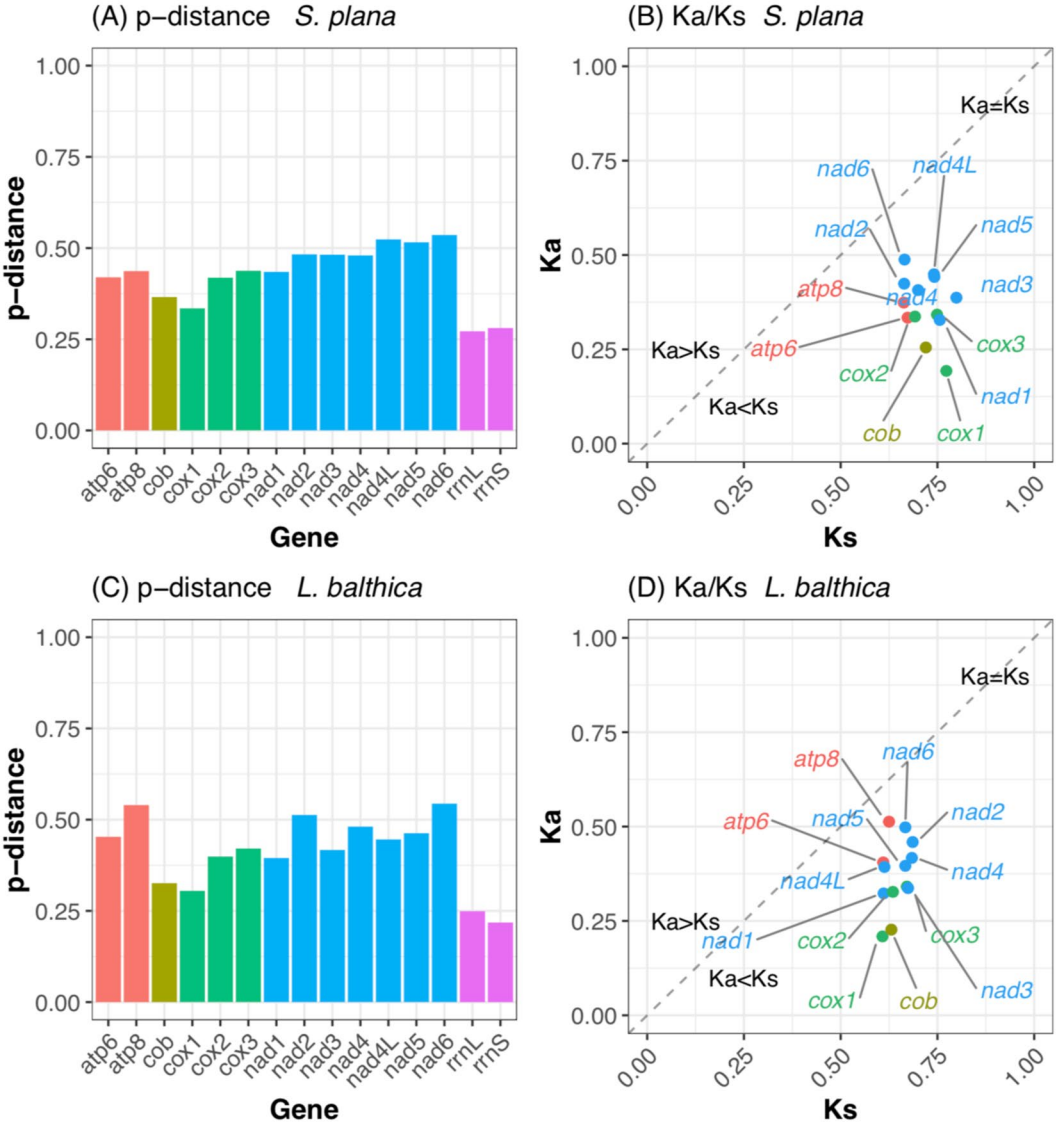
p-distances and rates of synonymous and nonsynonymous substitutions. Individual M-versus-F gene conservation expressed as p-distance and rates of synonymous and nonsynonymous substitutions within these same mt genes for *Scrobicularia plana* (**A,B**, respectively) and *Limecola balthica* (**C,D**, respectively). Genes are color-coded by gene family.

Genetic distances of individual genes between F and M mtDNAs were analyzed in each species. The results show that the level of conservation is higher for both ribosomal RNA genes (*rrnS* and *rrnL*), and also for the PCG *cox1*, whereas NADH dehydrogenase subunit genes (*nad* series) and *atp8* for *L. balthica* are generally less conserved (Fig. 2a,c). These results are consistent with the general findings in DUI bivalves^32^. For *S. plana* the average nucleotide divergence (measured as *p*-distance) of combined protein coding and ribosomal RNA genes is 0.43 ± 0.08 SD (based on values in Fig. 2a). For *L. balthica* the average *p*-distance of combined protein coding and ribosomal RNA genes is 0.41 ± 0.10 SD (based on values in Fig. 2c). The number of nonsynonymous substitutions per nonsynonymous sites (*Ka*) relative to the number of synonymous substitutions per synonymous sites (*Ks*) were also calculated (shown in Fig. 2b,d). This analysis provides an estimate of the degree of selection (either neutral, positive, or purifying) on each PCG. For both species and for all PCGs, all data points fall below the line representing neutrality (expressed as *Ka* = *Ks*; see dashed line in Fig. 2b,d), suggesting that the PCGs in *S. plana* and *L. balthica* accumulate more synonymous substitutions over evolutionary time relative to nonsynonymous substitutions. Therefore, all *Ka* and *Ks* rates supported an initial hypothesis of purifying selection for mitochondrial genes. To statistically test if the genes were indeed under purifying selection, we conducted a Z-test of Selection^52^. For all PCGs in both species, with the exception of *atp8* in *L. balthica*, there is strong statistical support (at α = 0.05) for rejecting a null hypothesis of neutrality (H_n_: *Ka* = *Ks*) in support of an alternate hypothesis for purifying selection (H_a_: *Ka* < *Ks*). This is because all pairwise Z-test p-values were <0.01 for both hypotheses. For *atp8* in *L. balthica* the Z-test of Selection did not support rejecting neutrality (p-value = 0.277 for H_n_: *Ka* = *Ks*), a result that might be affected by the small size of this gene (129 bp), which in turn altered the statistical power of the test. With regards to the large insertion within the male *cox2* gene for both species, these data are of even greater interest as they suggest that this insertion likely does not render the gene functionless. Rather, *cox2* (the alignable parts) actually remains among one of the more relatively conserved mt genes (Fig. 2). Overall, our results are in line with what has been observed in other bivalves with DUI, i.e. despite the considerable divergence of DNA and amino acid sequences of PCGs there is evidence of strong purifying selection acting on these mitochondrial genes^32^.

For comparative purpose, *p*-distances of individual genes (PCGs only) between F and M mtDNAs were also calculated for three species known for having the greatest F versus M DNA divergences in the families Mytilidae, Veneridae and Unionidae: i.e., the mytilid *Modiolus modiolus*^53^, the venerid *Ruditapes philippinarum*^17^, and the unionid *Quadrula quadrula*^14^ (Table 4). Our results show an average *p-*distance (for all 13 PCGs) of 0.45 for nucleotide (nt) and 0.53 for amino acid (aa) in *S. plana* and 0.44 (nt) and 0.53 (aa) in *L. balthica* (Table 4). To our knowledge, the nucleotide and amino acid divergences reported in *S. plana* and *L. balthica* are the greatest reported among DUI species, surpassing those in freshwater mussels (Table 4), which were previously thought to exhibit the greatest divergences between their sex-specific mtDNAs^14^. On the other hand, the average uncorrected nucleotide divergence observed between the F and M PCGs of the marine mussel *Mytilus edulis* is about 0.23^26^. This low level of divergence has been proposed to be a consequence of masculinization events, which are characterized by an invasion of the male route of inheritance by an F mtDNA that becomes transmitted through sperm as a standard M mtDNA^12,54^. These events reset the level of divergence between the F and M mitochondrial genomes to zero^12,54^. Conversely, the high level of divergence observed in freshwater mussels has been hypothesized to be a consequence of a complete absence of masculinization events for over 200 million years in Unionida^14,20^. According to these studies, masculinization would be no longer possible in this taxon because of the existence of the M-specific extension of the *cox2* gene, a specialized feature of the unionid M mtDNA that would prevent recombination between the F and M mtDNAs, i.e. a step necessary for masculinization to occur^12,14,20^. We thus propose that the high divergences observed between the F and M mtDNAs in *S. plana* and *L. balthica* are also related to an absence of masculinization events in these species because of the insertion in their M*cox2* gene (described below).

**Table 4:** Intraspecific divergence (uncorrected *p-*distances) of the 13 mitochondrial protein-coding genes between M and F mtDNAs in DUI species known for having the greatest F versus M DNA divergences. (a) nucleotides (b) amino acids.

	*cox1*	*nad4*	*nad3*	*nad4L*	*nad1*	*nad5*	*cob*	*cox2*	*atp8*	*nad6*	*atp6*	*cox3*	*nad2*	Average
**(a)**														
*Scrobicularia plana*	0.335	0.466	0.482	0.524	0.435	0.516	0.336	0.419	0.437	0.536	0.420	0.436	0.483	0.449
*Limecola balthica*	0.305	0.481	0.426	0.446	0.391	0.467	0.329	0.410	0.540	0.544	0.453	0.421	0.513	0.440
*Quadrula quadrula*	0.287	0.383	0.374	0.404	0.414	0.390	0.365	0.348	0.358	0.475	0.384	0.361	0.443	0.384
Modiolus modiolus*	0.280	0.375	0.373	—	—	—	—	—	—	—	0.466	0.375	—	0.374
*Ruditapes philippinarum*	0.222	0.300	0.278	0.468	0.240	0.299	0.281	0.336	—	0.313	0.298	0.274	0.347	0.305
(**b**)														
*Scrobicularia plana*	0.288	0.610	0.604	0.500	0.480	0.618	0.329	0.506	0.595	0.701	0.493	0.517	0.614	0.527
*Limecola balthica*	0.300	0.610	0.541	0.527	0.474	0.621	0.332	0.515	0.619	0.690	0.571	0.485	0.663	0.534
*Quadrula quadrula*	0.318	0.591	0.492	0.763	0.549	0.572	0.380	0.420	0.500	0.722	0.523	0.440	0.661	0.533
Modiolus modiolus*	0.233	0.445	0.449	—	—	—	—	—	—	—	0.576	0.422	—	0.425
*Ruditapes philippinarum*	0.123	0.334	0.303	0.779	0.216	0.346	0.273	0.419	—	0.560	0.343	0.314	0.444	0.371

NOTE – *The mtDNA sequence for *Modiolus modiolus is incomplete*.

### Mitochondrial ORFans

Unassigned regions in the coding strand were searched for the presence of supernumerary PCGs and mtORFans with a minimal length of 150 bp. Only ORFs encoding proteins with at least one predicted transmembrane domain were retained, because all sex-specific mtORFans characterized to date in DUI species (i.e. those encoding F-ORF in females and M-ORF in males), except for one case, possess at least one transmembrane domain or helix (TMH)^7,8,22,40^. In both *S. plana* and *L. balthica* F mtDNAs, only two unassigned regions were susceptible to contain supernumerary ORFs of >150 bp, i.e. between *rrnS-trnM* and *trnF-cox1* (Table 3). However, the region between *trnF* and *cox1* was discarded because of the presence of a possible 5′ extension of the *cox1* gene in all four genomes, an issue that will need to be assessed by looking at expression data. Otherwise, no ORFs corresponding to the expected profile were found in the F mtDNAs of *S. plana* and *L. balthica*. However, it is conceivable that smaller ORFs or ORFs encoding proteins without predicted transmembrane domain could be involved in DUI in these distantly-related species. The smallest F-ORF identified to date in a DUI species, i.e. in the unionid *Venustaconcha ellipsiformis*, is encoding an 89aa-long protein with one predicted TMH^7,40^, whereas the smallest M-ORF is potentially encoding a 30aa-long protein without TMH in *Mytilus californianus*^8^, although the functionality of this latter ORF remains to be demonstrated. Additional F mt sequences and expression data from *S. plana* and *L. balthica* will be necessary to clearly demonstrate the presence (or absence) of the F*-orf* gene in these species.

In both male mitochondrial DNAs, four unassigned regions contained supernumerary ORFs of >150 bp, i.e. between *cob-trnW*, *trnW-rrnS*, *trnG-cox2* and *rrnL-atp6* for *S. plana* and between *cob-cox2*, *cox2-trnW*, *trnG-rrnS* and *rrnS-trnM* for *L. balthica* (Table 3). Five ORFs corresponding to the expected profile were found in *S. plana*, two between *trnW-rrnS* and three between *trnG-cox2*, whereas four ORFs with the expected profile were found in *L. balthica*, one between *trnG-rrnS* and three between *rrnS-trnM* (Fig. S2). Sequence similarity searches using PSI-BLAST^55^ against non-redundant protein sequences and SWISSPROT databases failed to detect significant sequence similarity with known proteins for all these ORFs except one (i.e. mtORFan1 in the unassigned region *trnG-rrnS* of *L. balthica* M mtDNA; Fig. S2). For this sequence, our results revealed moderately significant hits (E-values 2e-07) with microtubule-associated proteins. This result is interesting since previous *in silico* analyses of M-ORF sequences in other DUI species also indicated connections with cytoskeleton proteins involved in microtubule-binding and actin-binding (e.g. ankyrin)^8,40^. These observations led to the hypothesis that M-ORF, with its predicted transmembrane domains, may target sites outside sperm mitochondria and be responsible for their cellular positioning in developing embryos^8^. Indeed, studies have shown that, after fertilization, only in male DUI embryos sperm mitochondria remain grouped together, and are eventually sequestered in the germ line, whereas they are dispersed and/or destroyed in female embryos (reviewed in Zouros^12^). M-ORFs are thus considered as ideal candidates for M mtDNA-derived masculinizing factors in DUI species^8,40^.

At this moment, however, we cannot confirm nor disprove the presence of a M-*orf* gene in the M mtDNAs of *S. plana* and *L. balthica*. Pairwise alignments between the ORFs found in both species were performed but it was not possible to obtain satisfactory alignments that could provide support for identifying a conserved ORF (data not shown). Again, additional M mt sequences and expression data from *S. plana* and *L. balthica* will be necessary to test for the presence of a M*-orf* gene in these species.

### Insertion in the male *cox2* gene

**Figure 3:**
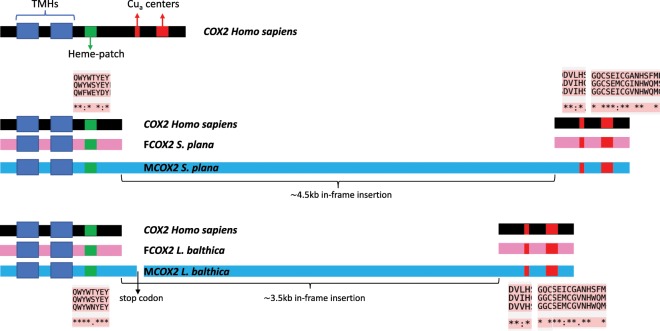
F*cox2* and M*cox2* structural features in *Scrobicularia plana* and *Limecola balthica*. For the sequence alignments of the “heme-patch” regions and Cu_a_ centers, identical amino acids are indicated by an *.

Annotations with MITOS revealed an insertion of >4.5 kb and >3.5 kb in the M*cox2* gene of *S. plana* and *L. balthica*, respectively, which is absent in the F*cox2* genes of both species (Figs. 1 and 3). In *S. plana*, this in-frame insertion, if translated, means that the *cox2* gene would be 5,679bp-long and, to our knowledge, would therefore encode for the longest *COX2* protein in the animal kingdom (i.e. 1,893 amino acids). The sequencing of additional *S. plana* male individuals revealed that this insertion is conserved among different individuals from different populations (Tassé *et al*. unpublished), indicating that it is most probably functional. A multiple sequence alignment of *Homo sapiens COX2* and *S. plana* F*COX2* and M*COX2* amino acids indicates that the insertion is situated between the “heme-patch” region, containing an important residue Trp that functions as the point of electron entry from Cytochrome C, and the first Cu_a_-binding center (Figs. 3 and S3), and *in silico* analyses suggest that it does not contain any additional transmembrane domain (Tassé *et al*. unpublished). In-frame insertions resulting in enlarged *cox2* genes have also been reported in hymenoptera, ciliates, brown algae and microflagellates^56^. As mentioned above, modifications to the *cox2* gene is a particular feature often found in DUI species. In unionid mussels, the longest *cox2* gene is found in the M mtDNA of *Hyridella menziesii* (1,380 bp)^22^ and is the result of a 3′ extension, as in other DUI unionids^21^. Previous studies indicated an extra-mitochondrial localization of M*COX2* as well as a possible involvement of the protein in reproduction in freshwater mussels^23,24^, further supporting the link between gender and mtDNA transmission patterns. In the order Venerida, i.e. the order to which *S. plana* and *L. balthica* belong, an in-frame insertion of 300 nt has been reported in the species *Meretrix lamarckii* (family Veneridae)^19^. However, such modifications of the *cox2* gene have not been reported in marine mytilid mussels, i.e. *Mytilus* spp., and in the venerid clam *Ruditapes philippinrum*, a duplicated copy of the *cox2* gene with a 3′ extension has been reported, but in the F mtDNA^12,19^.

The situation in *L. balthica* is quite different than in *S. plana* regarding the M*cox2* gene, which is split in two by an insertion. Specifically, this insertion divides M*cox2* into M*cox2a*, encoding the two transmembrane helices and the “heme-patch” region followed by a complete stop codon (TAA), and M*cox2b*, encoding an enlarged intermembrane space and the Cu_a_ centers (Figs. 3 and S4). This situation is confirmed by transcriptomic data (Illumina RNAseq reads from sperm cells, used to polish our nanopore reference mitogenomes), which indicate that both regions are transcribed (i.e. with discrete, non-overlapping M*cox2a* and M*cox2b* transcripts). Although it remains to be determined whether M*cox2a* and M*cox2b* are translated, it is worth noting that similar cases have been described in the freshwater mussel *Anodonta cygnea*^33^ and the Hymenoptera *Campsomeris* spp^56^. For example, a translocation of a portion of the *nad5* gene has been reported in *A. cygnea*, and since the translocated portion and the rest of the gene both possessed their own start codon, the authors proposed that they could be transcribed and translated separately^33^. In the genus *Campsomeris*, *cox2* is also split into two genes and this likely occurred through intragenic insertion of a cluster of several ORFs, one of which encodes a putative endonuclease that might have been directly involved in the process of *cox2* fission^56^. However, no ORFs with similarity to an endonuclease were found in the M*cox2* insertion of *L. balthica* (nor in *S. plana*). According to Szafranski *et al*.^56^, COXIIA and COXIIB polypeptides in *Campsomeris* spp. apparently assemble into a functional COXII heterodimer. Further studies will be needed to determine if this is also the case in *L. balthica*, or to clearly verify if a process of mitochondrial RNA splicing, because a group II intron has already been reported in the *cox1* gene of Annelida^57^, could be involved (i.e. M*cox2* RNA splicing or M*cox2a* and M*cox2b* RNA trans-splicing into a single mRNA).

It also remains to be determined if *L. balthica* possesses an enlarged M*COX2* or a M*COX2* protein of a typical length. For example, the stop codon in M*cox2a* could be read as a sense codon by a suppressor tRNA, i.e. a mutated (usually) tRNA that would insert an amino acid instead of initiating termination, as seen in several eukaryotes^58^. The presence of duplicated *trn* genes in the M mtDNA of *L. balthica* might be a clue for such scenario. Modification or duplication of tRNAs have already been proven to allow codon reassignment by changing codons from stop-to-sense or sense-to-sense in yeast, green algae and metazoan mitochondria^59,60^. Alternatively, termination can be avoided by ribosomal frame-shifting, a process that allows for protein merging from two or more overlapped ORFs^61^. This has been reported for the mitochondrial *cox1* gene of the diatom *Phaeodactylum tricornutum*: to avoid a stop codon, translational frameshift skips a nucleotide (called + 1)^62^. Similar strategies (+1 or −1 frameshifts) have also been recorded in metazoan mitochondrial genomes including ants, turtles and humans^63–65^. Further transcriptomic and proteomic analyses, and a closest look in mitochondrial tRNAs, will be required to validate any machinery involved in M*cox2* transcription and translation in both *L. balthica* and *S. plana*. The results presented in this study clearly indicate that the relationship between *cox2* variations and DUI deserves much greater attention.

## Conclusion

In summary, the description and comprehensive analysis of complete F and M mtDNAs of two newly discovered bivalve species with DUI from two additional families (Semelidae and Tellinidae), have led to new insights into DUI mitogenomics. Our results revealed uncorrected amino acid *p-*distance of ~53% for PCGs between the M and F genomes of both species: this is the highest divergences reported in DUI species thus far. Hence the way (or ways) in which males of bivalves with DUI can tolerate heteroplasmy characterized by such high variability remains an important unanswered question (e.g. see Bettinazzi *et al*.^66^). Our results also highlighted an extremely unusual feature of the M genomes of *S. plana* and *L. balthica* compared to their female-transmitted counterparts. This feature is the presence of an important in-frame insertion (>3.5 to 4.5 kb) in the M*cox2* gene. This is the longest insertion reported in the Kingdom Animalia, which remains to be functionally characterized. The reported data further indicate that the newly-sequenced M mitogenomes may be carrying lineage-specific genes (mtORFans) possibly involved in the DUI process. Analyses of complete mtDNAs from additional bivalve species and further protein-based studies are needed to elucidate the number, taxonomic distribution, evolution, and function of mtORFans and atypical *cox2* genes in this group, as well as the molecular mechanisms underlying DUI.

## Methods

### Specimen collection and sample preparation

Adult specimens of *Limecola balthica* were collected at low tide on the Aytré sandy mudflat (France; 46.1259 N, 1.1284 W) in April 2016 and May 2017 (these are periods during which gonads are known to be near sexual maturation^42^. Individuals were carefully opened and gonad tissue nicked with a micro-scalpel. Gametes were then sampled with a micropipette and a small amount was used to sex the animal with a light microscope; the remainder of the sample was flash-frozen in liquid nitrogen and stored at −80 °C. Adult specimens of *Scrobicularia plana* were collected in May 2013 from Concarneau (France; 47.8728°N, 3.9207°W). Individuals were dissected and gonads inspected under the microscope. Female and male mature gonads were conserved in 95% Ethanol and sent to the Université de Montréal.

For *S. plana*, total genomic DNA was extracted separately from male and female gonad tissues with a Qiagen DNeasy Blood & Tissue Kit (QIAGEN Inc., Valencia, CA) using the kit provided animal tissue protocol. The quality and quantity of DNA were assessed by electrophoresis on a 1% agarose gel and also with a BioDrop µLITE spectrophotometer. One female and one male gonad sample with the highest purity and concentration were chosen for sequencing. For *L. balthica*, total genomic DNA from male gonadal tissues (majority of gametic cells, and some somatic carry-over) was extracted using the phenol-chloroform protocol described in^67^, resuspended in molecular grade water, and quantified using a Nanodrop 2000 and a Qubit fluorometer (dsDNA HS assay kit, Molecular Probes). DNA integrity was also checked by electrophoresis on a 1% agarose gel. One sample characterised by the highest purity and concentration was chosen for sequencing.

### Mitochondrial genome sequencing

Total DNA extractions from *S. plana* male and female gonads were respectively used to prepare two DNA libraries that were paired-end sequenced (2 × 100 bp) using an Illumina HiSeq platform (Génome Québec Innovation Centre, Montréal, QC, Canada). Paired reads were trimmed with Trimmomatic 0.32^68^ and merged with Pear 0.9.6^69^ on the Galaxy online platform^70^, checking the quality of reads at every step with FastQC 0.11.5^71^. MITObim 1.8^72^ was used to assemble the complete genome sequence, starting from a *S. plana* partial *cox1* sequence (GenBank accession number KX447421 for the male mtDNA and KX447423 for the female mtDNA) as an initial seed.

For *L. balthica*, the library preparation was done following the SQK-LSK108. Nanopore protocol. A DNA shearing step (using Covaris gTubes) was included to linearize circular mtDNA molecules (1 min at 11k RPM in a 5415 R Eppendorf centrifuge). The resulting library was sequenced on the Minion MIN-101B sequencer using a R9 flowcell. Basecalling was performed using Albacore 2.1.3^73^, followed by quality control using Minion_QC 1.0^74^. Nanopore adapters were removed and chimeric reads were discarded using Porechop 0.2.3 (Wick R. Porechop, available at: https://github.com/rrwick/Porechop). Reads with quality scores <9 were removed using Nanofilt 2.0.0^75^. Cleaned reads were assembled using Canu 1.6^76^ considering an approximate genome size of 2 Gb and disregarding all reads smaller than 500 bp. Resulting unitigs were blasted locally using blastn^55^, using male and female *rrnL* sequences (Genbank accession numbers KX831969 and KX831970, respectively)^42^. Based on these results, we identified tig00000009 (length of 26,120 bp, assembly based on 61 nanopore reads) as the male mitogenome. We did not retrieve the female mitochondrial genome as one contig but as two (tig00000888: 16,790 bp, 34 reads; tig00000885: 6,319 bp, 1 read). Basecalling errors were corrected using signal-level data with Nanopolish 0.9.0^77^. We used these two cleaned contigs as reference genomes to map Illumina 2 × 150 bp HiSeq sequences (data quality control and pre-processing step as in^78^) produced from a second individual using Pilon 1.21^79^. HiSeq sequencing of cDNA was performed based on mRNAs purified from sperm and foot tissue (for mapping onto the male and female mitogenomes, respectively) using the Macherey-Nagel Nucleospin RNA kit for NucleoZOL (RNA quality control, cDNA synthesis, library preparation, sequencing, data quality control and pre-processing as in^80^). By mapping high-quality Illumina reads on our draft nanopore contigs, we were able to further correct sequencing errors. The two F-type contigs (tig00000885 and tig00000888) were then merged. The quality of the mitogenome assembly was checked at each step of the pipeline (initial Canu assembly, Nanopolish polishing, Pilon polishing) by calculating the percent identity between our F-type unitigs and a published F-type mitogenome from the same sampling locality (KM373201)^43^ using MUMmer 3.23^81^.

Mitochondrial genome sequences were deposited in GenBank (accession codes MN528026 and MN528027 for the F and M mtDNAs of *S. plana*, respectively, and accession codes MN528028 and MN528029 for the F and M mtDNAs of *L. balthica*, respectively).

### Characterization of mtDNAs and sequence analyses

The male and female mitogenome sequences obtained were annotated with MITOS2^82^. Protein-coding genes (PCG) were also predicted using NCBI′s Open Reading Frame Finder, choosing the invertebrate mitochondrial genetic code and alternative start codons. Candidate transfer RNA (tRNA) genes were also identified using tRNAScan-SE^83^. The ends of 16S and 12S rRNA genes were assumed to extend to the boundaries of their flanking genes. The complete mitochondrial genomes, with their annotated PCG, tRNAs and rRNAs, were illustrated with GenomeVx online tool^84^.

The degree of genetic conservation among the M-versus-F genes in each species was assessed using two approaches: (i) pairwise *p*-distances, and (ii) the rates of synonymous and non-synonymous substitutions, i.e. the number of synonymous substitutions per synonymous sites [*Ks*] and number of non-synonymous substitutions per non-synonymous sites [*Ka*], which were calculated in MEGA v7^52^. Since there were various indels present between the individual M- and F-type genes within both species, pairwise deletion was used to account for gaps/missing data-points and codon-alignments were used to facilitate accurate calculations of genetic distances, as well as *Ka* and *Ks* values. Amino acid sequences were determined using EMBOSS Transeq and by assuming an invertebrate mitochondrial code^85^. Codon-alignments were generated by aligning M and F amino acid sequences for each gene using MUSCLE^86^ in the online portal found at http://www.ebi.ac.uk/Tools/msa/muscle/84. Using these protein alignments as references, nucleotide sequences for each respective gene were then codon-aligned via PAL2NAL v14^87^. The exceptionally large indel within the M*cox2* gene was manually excluded from the gene alignments. MEGA was also used to conduct a Z-test of Selection [via the Nei-Gojobori method] for each gene^52,88^. All plots relating to gene conservation were generated in R version 3.4.1 (R Core Team 2017) using the package ggplot2 v3.2.0^89^.

For comparative purpose, *p*-distances of individual genes between F and M mtDNAs were also calculated for three species known for having the greatest F versus M DNA divergences in the families Mytilidae, Veneridae and Unionidae: i.e. the mytilid *Modiolus modiolus* (GenBank accession: F, KX821782; M, KX821783)^53^, the venerid *Ruditapes philippinarum* (GenBank accession: F, AB065375; M, AB065374)^17^, and the unionid *Quadrula quadrula* (GenBank accession: F, FJ809750; M, FJ809751)^14^. These comparisons facilitated a better understanding of the selection pressures acting on the PCGs in these species. Finally, the presence of supernumerary mitochondrial protein-coding genes (and mtORFans) in unassigned regions was examined using ORF Finder (NCBI). We retained only ORFs with a minimal length of 150 bp and localized on the coding strand, as mitochondrial genes are only encoded on one strand in our two species. Transmembrane domains were predicted using the TMHMM server v2.0 (http://www.cbs.dtu.dk/services/TMHMM/). Alignments between predicted ORFs were done with MUSCLE^86^ and the percentage of identity was visualized with MView^90^, via the EMBL-EBI online web services^85^ keeping default parameters.

## Supplementary information

Supporting Information.
