# Supplementary material for: Unorthodox features in two venerid bivalves with doubly uniparental inheritance of mitochondria
Source: Sci Rep. 2020 Jan 23;10:1087. doi: 10.1038/s41598-020-57975-y (PMC6978325; doi:10.1038/s41598-020-57975-y)
Supplement: Supplementary file 1 — Supporting Information. [file 41598_2020_57975_MOESM1_ESM.pdf]

Unorthodox features in two venerid bivalves with doubly uniparental inheritance of mitochondria

Charlotte Capt[1,a], Karim Bouvet[1], Davide Guerra[1], Brent M. Robicheau[2], Donald T. Stewart[3], Eric Pante[4,*] Sophie Breton[1,*]

[1] Department of Biological Sciences, Université de Montréal, Montréal, QC, Canada

[2] Department of Biology, Dalhousie University, Halifax, NS, Canada

[3] Department of Biology, Acadia University, Wolfville, NS B4P 2R6 Canada

[4] Littoral, Environnement et Sociétés (LIENSs), UMR 7266 CNRS–La Rochelle Université, 2 rue Olympe de Gouges, 17000 La Rochelle, France

Figure S1. Secondary structures of trnP and trnA duplications in the M mtDNA of L. balthica.

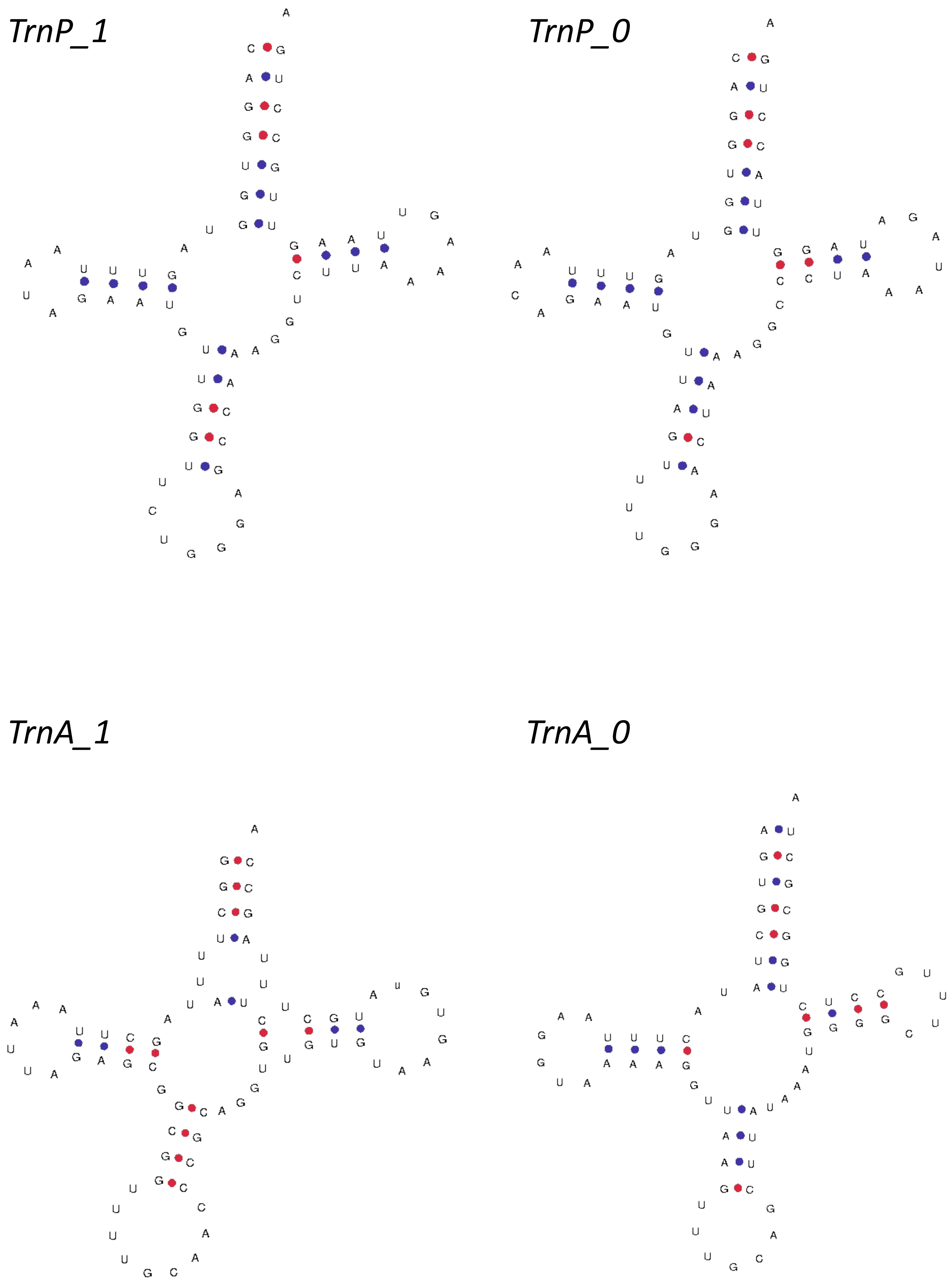

Figure S2. Sequence and secondary structure of predicted ORFs found in all unassigned regions longer than 150nt per sex per mitochondrial genome. Only ORFs with at least one predicted TMH were retained.

Scrobicularia plana M mtDNA

Unassigned region trnW-rrnS

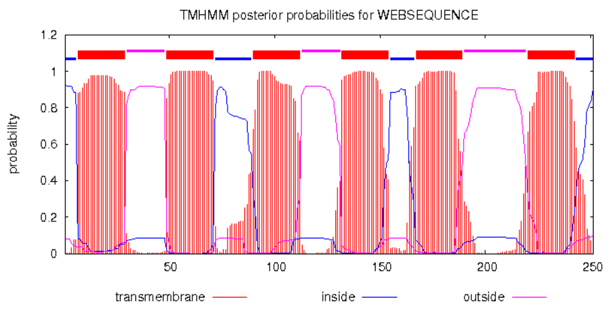

mtORFan1
MECALMAVYVFAEWLWVLTGLNFCSSRVLFSLSSSKGPDM
FWLSSTNRLCVFVVSGFSSIGSILVCVICFLVKSRYSVALASI
CPASLYYSLCLYVLALTALYCCLLWEIMIRDCPDDSFSKMLI
NHTYTPVYLMGSFSVMLSTFLFSGNFCSVKLGWFMSDSLF
GFILGVIYLSSALSLISWVFLTFLMMFYSYDTMDKYPDTER
EWRFVVSEISWAGFIWGFILTMLCVCFSLILFSVLGVGGFVP
SFE

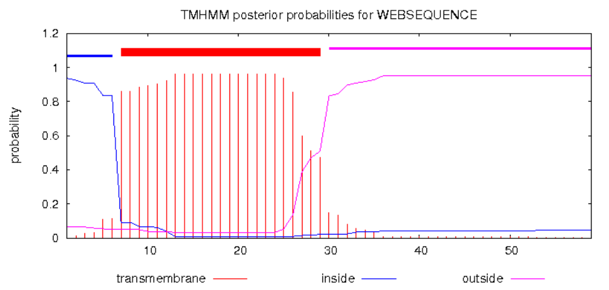

mtORFan2
MGGYRFVVGSVSTLVVTGLCLPALAGSGSYFSGSVDWPSL
KSLALALGWSCLHSDTMVL

Unassigned region trnG-cox2

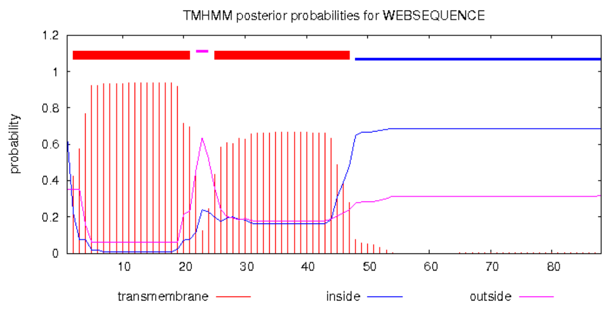

mtORFan3
WALLVFYFTFVLSTWCLFIRAYKMLLVMVEFYLSSASVLG
LSLLSSVKPTLIRKPESFVRILKLQESPSLVSLVKGSFEVNM
GLAVLG

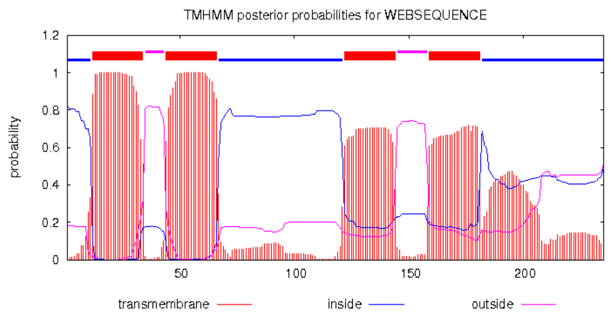

mtORFan4
MVGFAFGSGTGYFMLGAMIVFMLLAFGFALVYFHKSAASG
DWSLSVLGLVLSLSSVLLVLTACFCLFKGGMNVVNSGEMVC
GGIGSFGEPVVGAMNSKGAMGLVSIIKVPSDYSEFKSIGGFL
LYKVLMNSGLAAECGGWLFGPGVKMMLTPSLTTDLTGIYW
TSSVSETFFFFTARVVIADVLWGVGKFAGAVLGVVDACLGHP
FAELMPTVNIPYKNLLMNILLVASELIMYL

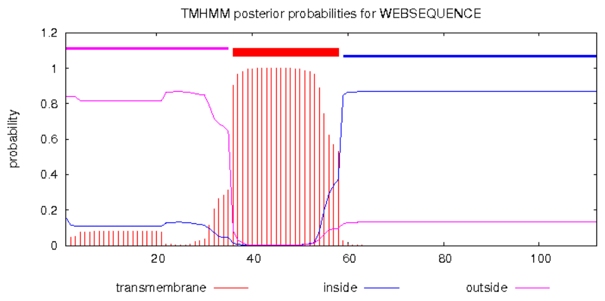

mtORFan5
LSLLWMMNMCVEFSVFNSWWVQNTKLSLGNWALCDVVGIA
GFLFYFCFIYVVLVYPSLQDIASNGRVLFKKGEGVSAKPIKEGE
TYIDPETGEVCTDPETSGVPKPREVSKS

Limecola balthica M mtDNA

Unassigned region trnG-rrnS

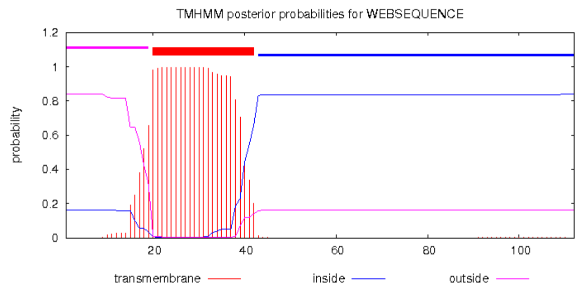

mtORFan1
MFMNVSMSDSFFDGDLSLIFLSFIFFSLVVGCGSQLLIFT
WSSPEKAQEEILQEEILQEEILQEVTLQGEILQEEMLQEET
LQEETLQEEMLQGETGVSGGGFLIRKVLIMM

Unassigned region rrnS-trnM

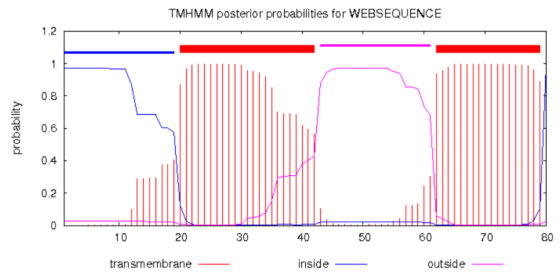

mtORFan2
VGMHYFESSSVGSMIRLGGYALVLCWMVMLELISLGDVIS
YLVGETLGDLVLHHVGVPELEFNHYAFIFYMMVFFNIWWM

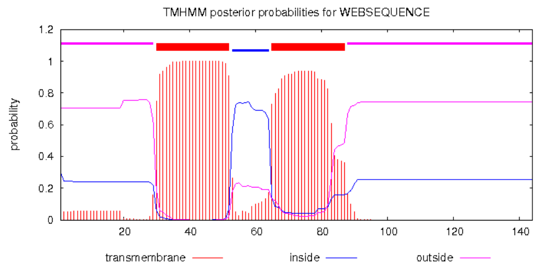

mtORFan3
RIYSSIAGACIIIVREEVFKISSFAEDPHFISVCILSLMLI
VLSVICFFVFFGSEIVKLVSTSKFVKGFLSTLFSAFLRF
YFMQGYFFGTPEASEPRVLPSGHKDPNKTPKKSPKK
GPKKSPKGPRGGNEGSGSSKGARRKLKF

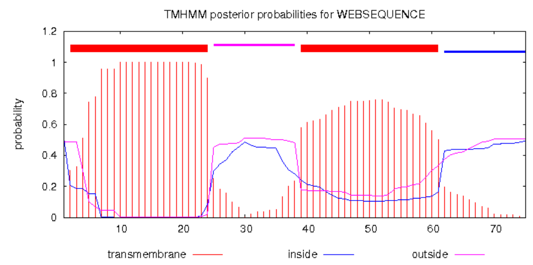

mtORFan4
SGFLGHFLGLFYVSILCSAIFLVLRKLLSPEFYHRGMSIL
MSLLSSVLSSVLSNPLSGLVGGTSAGVGLKVPVVS

Figure S3. Scrobicularia plana F and M COX2 proteins alignment. Identical amino acids are indicated by an *.

Alignment of *COX2*: *H. sapiens* vs *S. plana* F*COX2* and M*COX2*

MSA

The multiple sequence alignment result as produced by T-coffee.

```
T-COFFEE, Version_11.00.d625267 (2016-01-11 15:25:41 - Revision d625267 - Build 507)
Cedric Notredame
SCORE=921
*
 BAD AVG GOOD
*
Hsapiens     :  91
Fcox2        :  90
Mcox2        :  84
cons         :  92

Hsapiens     MAHAAQVGLQDATSP-------------IMEELITFHDHALMIIFLICFLVLYALFLTLT------TKLTNT
Fcox2        -----------------------------MLGLIFLHDYLMCMCFMIMSVVGSVLFLVVPKVSYFCGGIHFR
Mcox2        MNEVTSQGVNPRLRFVVDASVSPDWANSEGLDLILLHDYLMLFCFFIMSAMLMGVYYNFGKKGLMVIRGMYR

cons                                         ** :**: : : *:*   :   ::: .

Hsapiens     NISDAQEMETVWTILPAIILVLIALPSLRILYMTDEVNDP-SLTIKSIGHQWYWTYEYTD-YGGL-------
Fcox2        NVYRNNSLELWWTIIPIFLIIIMGYPSFVQLYAMGMNDKPKFITVKITGHQWYWSYEYLVHFPSLVSLAEEL
Mcox2        AVRNKQMLEFSAAIFAILLVIMMGYPSFVKLYSMGMNDTEKSMSLKAVGHQWFWEYDYFVDMENLLELCDKY

cons          :   : :*   :*:. ::::::. **:  **  .  :    :::*  ****:* *:*     .*

Hsapiens     ------------------------------------------------------------------------
Fcox2        GVFKEYNQ----------------------------------------------------------------
Mcox2        KVFPHWEGYGKKSLGDEGYALFDDSGPKTETVKDIVGDEEPEILASVFDLVLFDDSGPKTETVKDIVGDEEP

cons

Hsapiens     ------------------------------------------------------------------------
Fcox2        ------------------------------------------------------------------------
Mcox2        EILASFFGECACLEAEMVKGGVGFSLNTYMSPPICDGKSSAPGVKGQPGSGAEGAPTPADDPRPKGKSLPKP

cons

Hsapiens     ------------------------------------------------------------------------
Fcox2        ------------------------------------------------------------------------
Mcox2        VMYDPDVDYRNLPPCPGPKKRNRPVVETDIKNLPWSARRLLFKEVKEQSCREMLGTGPEGDSSQQKGVPRLS

cons

Hsapiens     ------------------------------------------------------------------------
Fcox2        ------------------------------------------------------------------------
Mcox2        VLAARALPGDVVGDGDCPPQNPGNNVGTPPVQGDGAVGLNPAVQDPVAMVLNNPAPAPADAAENLAAEAPAG

cons

Hsapiens     ------------------------------------------------------------------------
Fcox2        ------------------------------------------------------------------------
Mcox2        GEGNAPVQEPAQAVLPSTPPRSGMEEMPSILLKKKDPQLCPKFRGGPYKPRCAKQLDFNNPPHPPVPPFIPH

cons

Hsapiens     ------------------------------------------------------------------------
Fcox2        ------------------------------------------------------------------------
Mcox2        YLQNNLQGYIYPWVPVVGNNLGGGQGQAGVQGQVGGQVQVGGQAPAGGQVQVGGQAPVGGQVQVGGQAPAGG

cons
```

```
Hsapiens    ------------------------------------------------------------------------
Fcox2       -----GFTKGSGSS----------------------------------------------------------
Mcox2       QGQGAGVDNGSGVDEDMSQCTRGMSGEGDVDGSGPTMLVDKSLPSGLYLKKCEGGYNLGKGVDLLKVLKKVH

cons

Hsapiens    ------------------------------------------------------------------------
Fcox2       ------------------------------------------------------------------------
Mcox2       PEFGSGPRGEGPSNDGPQPQGMGPLEFLYIFWCEGCIWYSKLNLAFFVQDLWAVNRSWGRGHAVNPELFSSA

cons

Hsapiens    ------------------------------------------------------------------------
Fcox2       ------------------------------------------------------------------------
Mcox2       DKSFMCLWRMPLVSKVIMPLWHNLGLASQVSVEYHNDPSVLRVVTVLSPGPDNSVYMKVIYNQGVNLESLNF

cons

Hsapiens    ------------------------------------------------------------------------
Fcox2       ------------------------------------------------------------------------
Mcox2       SKSMMFQGLSVSGLNSDIKVALIWLVKKGGPEDINGPNRPSRPFKGPSVGGGTSKSDPFAGLTNRPMDPSPE

cons

Hsapiens    ------------------------------------------------------------------------
Fcox2       ----------------------EES-----------------------------------------------
Mcox2       RPVQDEPSNGGSGAQGGAEGSTQESEWSEGSYDGGTGRADRCLLDPSPKRSGGEAGPKEPQPADEAGEQEPS

cons

Hsapiens    ------------------------------------------------------------------------
Fcox2       ------------------------------------------------------------------------
Mcox2       LPEGSNTSVSDLSNCEFPSGLNLVFSGASNNLDDKPLGAAVYDGNGNMVIDENNNLIMIELSDEEEDMEGVE

cons

Hsapiens    ------------------------------------------------------------------------
Fcox2       ------------------------------------------------------------------------
Mcox2       VNNPSSQLGSGFALSFIFEMMDPFYDPSEHEDQVVPEATIVEMPDPKTGSQLVPEEELPAPEPVQEIEPLPD

cons

Hsapiens    ------------------------------------------------------------------------
Fcox2       ------------------------------------------------------------------------
Mcox2       ESRSEDIPRPSAQCISSFFDFFMKVFCFFMSSPFLGISCFPFGDFNPWDIDYALLGAGSVMGGYDSLYEDGF

cons

Hsapiens    ------------------------------------------------------------------------
Fcox2       ------------------------------------------------------------------------
Mcox2       KLFMDTGGPKKNSSPGGPEQGSSGESRALLPKNSSVKKTYGSLGNTPPFPPHTPDHSKVTEPLPGTVVPKSY

cons

Hsapiens    ------------------------------------------------------------------------
Fcox2       ------------------------------------------------------------------------
Mcox2       IMGALDWAYKKLLRPRVKSHPPILPGTSKMGQSGEAPLRPSPSRVKTMVDDCVVPTISKKLLFNRNMVKDYP

cons
```

```
Hsapiens    ------------------------------------------------------------------------
Fcox2       ------------------------------------------------------------------------
Mcox2       LNFPTLKSGNKPIYVISQRRSPSPSQPSLESSTPNLAYKSLFMGPPKKDVVDQGVQTSDSEHGSESSGTSGR

cons        


Hsapiens    ------------------------------------------------------------------------
Fcox2       ------------------------------------------------------------------------
Mcox2       GHYLSTFEIMETLRASAAQSSTPIPGAPMPKNLDRIMEILNTSGEGIWDEEARQEAEAFMDDDSWRGHNLEL

cons        


Hsapiens    ------------------------------------------------------------------------
Fcox2       ------------------------------------------------------------------------
Mcox2       HGVIDSDPLLEALSEIGFDEVDRRSLNSEQTLSVASISGKVKFISLDDTRTNIYWYTLVVDSSDDMTGGGRD

cons        


Hsapiens    ------------------------------------------------------------------------
Fcox2       ------------------------------------------------------------------------
Mcox2       DNSGGGSNRSLVLENTNDNAKSGSKGFNPPSLKSSKGKLGMREYVIKSFSISFLGGGCCQKFDSSSSPMEVI

cons        


Hsapiens    ------------------------------------------------------------------------
Fcox2       -------------------------------------------------------------------SIDF-
Mcox2       SEFEWRVEEVLCSEEMKVYIKSVAGSKLNGSRESMDRMIAEDMVNSNLFHYFFNSGESAPNSLASSSVYEFN

cons        


Hsapiens    ------------------------------------------------------------------------
Fcox2       ------------------------------------------------------------------------
Mcox2       PCKDSSESLSFVGSVSIDQRNVYVPVPLWGQYRSWGGKQSLSSGSVGDLTSLFPKGNQDSISGVSGQVAPPR

cons        


Hsapiens    -----------------------------------------IFNSYMLPPL--FLEPGDLRLLDVDNRVVLP
Fcox2       ---------------------------------GNVSDWLISYDSYTITASDSSMDFGYRYGQYVDYPMVLP
Mcox2       SPRRPRGSSLVASRYSSDSNGLGSAESGLFSNVFFTKAWLISYDSYTVPAESMADVPKFKYGQTVDYPAVLM

cons                                                  ::** :..              **   **


Hsapiens    IEAPIRMMITSQDVLHSWAVPTLGLKTDAIPGRLNQTTFTATRPGV-YYGQCSEICGANHSFMPIVLELIPL
Fcox2       GDSNVEVKVTSADVIHCWTIHGLGVKMDAVPGRVNTSHLANLRPGFAGWGGCSEMCGINHWQMSAEVEVLSV
Mcox2       GDSNVEVMVTSSDVIHSWTVTGLGVKIDAIPGRLNAVHLRNLRPGFTSWGGCSEICGVNHWQMGVEVEVLSP

cons         :: :.: :** **:*.*::  **:* **:***:*   :   ***.  :* ***:** **  *   :*::.


Hsapiens    KIFEMGPVFTL---------
Fcox2       EDFILWILTWVYSDMKDE-E
Mcox2       RDFLLWLLMMVFLDMSSLDE

cons        . * :  :  :
```

Figure S4. Limecola balthica F and M COX2 proteins alignment. Identical amino acids are indicated by an *.

Alignment of *COX2*: *H. sapiens* vs. *L. balthica* F*COX2* and M*COX2a*

MSA

The multiple sequence alignment result as produced by T-coffee.

```
T-COFFEE, Version_11.00.d625267 (2016-01-11 15:25:41 - Revision d625267 - Build 507)
Cedric Notredame
SCORE=848
*
 BAD AVG GOOD
*
Hsapiens     :  88
Fcox2Lbal    :  89
Mcox2aLbal   :  86
cons         :  84

Hsapiens     MAHAAQVGLQDATSPIMEELITFHDHALMIIFLICFLVLYAL------FLTLTTKLTNTNISDAQEMET
Fcox2Lbal    MFG---FSISSRL------------PCLCVFYNHISCSKCAFFLVVPKVSYFCGGIHFRNVYRNNSLEL
Mcox2aLbal   MKE---LCVENWGSPKGKEMVLLHDYFVLFGFVIMAIIFTVLVVYLRKGGLMGTRGMYRNVRVNQGLEF

cons         *     . :..                : . :        .:         :       *:   : :*


Hsapiens     VWTILPAIILVLIALPSLRILYMTDEVNDPS-LTIKSIGHQWYWTYEYTDYG-GLI------------
Fcox2Lbal    WWTIIPILLIIIMGYPSFVQLYAMGMNDKPKFITVKVTGHQWYWSYEYLVHLPSLISLADELGVFNNYN
Mcox2aLbal   SWSVLPMVLLVCMGYPSFCSLYDGSLGDSPKSMGLKVTGHQWYWNYEYFVNIEELVDQCLKTKALESAK

cons          *:::* :::: :.  **:  **  .  :.*. : :*  ******.***      *:


Hsapiens     -------------------------FNSYMLPPL--FLEPGDLRLLDVDNRVVLPIEAPIRMMITSQDVL
Fcox2Lbal    NQGASKGELEANVQFEGVSDWLISYDSYTVNNVDGVLESGFRYGQFVDYPMVLPGDSNVEVKVTSADVI
Mcox2aLbal   GPGVLANSGPKKETLEGVIEG-----------------------------------AEAEAAPMSSSAK

cons                                                         :  .    * ..


Hsapiens     HSWAVPTLGLKTDAIPGRLNQTTFTATRPGVY-YGQCSEICGANHSFMPIVLELIPLKIFEMGPVFTL-
Fcox2Lbal    HCWTIQGLGVKMDAVPGRVNSAHFSDLRPGFSAWGGCSEMCGVNHWQMSAEVEVLTVSDFILWILTWVY
Mcox2aLbal   GPGVLANSGPKKETLEGVIEGLSQSLLR-----------------------------------------

cons            .:   * * ::: * ::    :  *


Hsapiens     -------
Fcox2Lbal    SDIKDLD
Mcox2aLbal   -------

cons
```

Alignment of *COX2*: *H. sapiens* vs. *L. balthica* FCOX2 and M*COX2b*

MSA

The multiple sequence alignment result as produced by T-coffee.

```
T-COFFEE, Version_11.00.d625267 (2016-01-11 15:25:41 - Revision d625267 - Build 507
Cedric Notredame
SCORE=845
*
 BAD AVG GOOD
*
Hsapiens     :  79
Fcox2Lbal    :  75
Mcox2Lbal    :  44
cons         :  84

Hsapiens    MAHAAQV-GLQDATSPIMEELI------------------------------------------------
Fcox2Lbal   MFGFSI----------------------------------------------------------------
Mcox2Lbal   MSGSAKGPGVLANSGPKKETLEGVIEGAEAEAAPMSSSAKGPGVLANSGPKKETLEGVIEGAEAEVAPM

cons        *   :


Hsapiens    ------------TFHDHALMIIFLICFLVLYALFLTLTTKLTNTN-------------------------
Fcox2Lbal   --SS----RLPCLCVFYNHISCSKCAFFLVVPKVSYFCGGIHFRN-------------------------
Mcox2Lbal   SSSAKGPGVLAKSCALVNVGSNKGGVLGQSLEKTMASCG-LHVRSEGSSGSSSPGCKEEWNCKVWQAGS

cons                                  :             :   .


Hsapiens    ----------------------------------------------------------ISDAQ-------
Fcox2Lbal   ----------------------------------------------------------VYRNN-------
Mcox2Lbal   RAGSAEQPAKAWTWSGSSWIVKAEGDKSRVFTCFESVDGKTRYKANYPVDENNNNFMGVLNNNECFSFL

cons                                                          :   :


Hsapiens    ----------------------------------------------------------EME---------
Fcox2Lbal   ----------------------------------------------------------SLE---------
Mcox2Lbal   GHNSVVEPVEGLDLPEPGLPKPNLPGDDNPISGSGTSSKCSSEAVGHKEGSSLLEHNNIVEPVEGPDLP

cons                                                           :*


Hsapiens    ----------------------------------------------------------------------
Fcox2Lbal   ----------------------------------------------------------------------
Mcox2Lbal   EPGLPKPNLPGDDSPVSGSGTSSGCSPLVGGDGAGYKAEGSVKSEWGPDGELQLAVDKSSSSFNVKEGD

cons


Hsapiens    -----------------------------TVWTILPAIIL------------------------------
Fcox2Lbal   -----------------------------LWWTIIPILLI------------------------------
Mcox2Lbal   DMSGSRECGGPWVSKAGNFSPYSCTFFGGSWWSFWVIHCMDNGGDANPVGGTGSGGLDGTPDQDNAENE

cons                                       *::     :


Hsapiens    ----------------------------------------------------------------------
Fcox2Lbal   ----------------------------------------------------------------------
Mcox2Lbal   DSNAGGGGNGDSDLRNDHEDPHGDGLVAWMLNFFIHAVATIFGFAPNPDRIWETLIHPRRSNETILAEV

cons


Hsapiens    ---------------------------------------------------VLIALPS------------
Fcox2Lbal   ---------------------------------------------------IIMGYPS------------
Mcox2Lbal   GTPVDVCGGQGFSGWFTCKEWRPVGLYLTVDGRMSGGSLVNSGNLSPCSRYGCMNYPMPGSENRVPVEF

cons                                                         :  *


Hsapiens    -------------------------LRILYMTDEVND--PS-LTIKSIGHQWYWTY--------------
Fcox2Lbal   -------------------------FVQLYAMGMNDK--PKFITVKVTGHQWYWSY--------------
Mcox2Lbal   GEWTVIFEFLTHSIPGLVGTPLPPMFVMPFDQSTHGSVGSAASTASSESHWNYWSTGGYFVGPGLPNVS

cons                         :   :  .  ..  .   * .  .*  **:
```

```
Hsapiens     ---------------------------------------------------------------------
Fcox2Lbal    ---------------------------------------------------------------------
Mcox2Lbal    TPSLTPVFSSLAATTDSDSEMNGISPINPLNQVSLMWDPEYNYDEFVNLTVSRVFPDRDGVSAEVSFAE

cons         

Hsapiens     ---------------------------------------------------------------------
Fcox2Lbal    ---------------------------------------------------------------------
Mcox2Lbal    RQLFRNFLDNMFCVEGVNTVRMVPSQISVVPSSVYLAPSGTSLTNSTTSNESFLGSIGAVISNGASAGE

cons         

Hsapiens     ----------------------------------------------EYT--------------------
Fcox2Lbal    ----------------------------------------------EYLVHL-----------------
Mcox2Lbal    LSIAHRPEPFADSVAVEDGEVPGTSSTPEGEEGAVGGLVSQRKKEYEALVDLGKSGKILPKDLPKKYSS

cons                                                       *

Hsapiens     ---------------------------------------------------------------------
Fcox2Lbal    -PSLISLADELGVFNNYNNQG------------------------------------------------
Mcox2Lbal    APTLTNMIRLRGIFSGSNINQSDTSVFHPRFVSGPTDSKSAPAISESPNRGNSVGPQADPSSVKPVNIN

cons         

Hsapiens     ---------------------------------------------------------------------
Fcox2Lbal    ---------------------------------------------------------------------
Mcox2Lbal    SAPSIPGGPDRGNSIGPQADPSSVKPVNINSAPSIPGGLDRGNSVGPQADPINVEPVSVNSAPALSEGF

cons         

Hsapiens     ---------------------------------------------------------------------
Fcox2Lbal    -----------------------------------------ASKG------------------------
Mcox2Lbal    NLGNTIDFQPGASNVKPVVKDQAVPSNTVLDGVVNSTNKVESDDGSHGASLVDNAQKKGKKVKKKAVVV

cons         

Hsapiens     ---------------------------------------------------------------------
Fcox2Lbal    -----------------------------------------------------------------ELEA
Mcox2Lbal    KFWDKRHTFKLGRHKPSDGGSGPDRGSVVGPQPGPSGVKTMVKGQTGPSNKVVDSDSNTESDDGGQGGA

cons         

Hsapiens     ---------------------------------------------------------------------
Fcox2Lbal    NVQ------------------------------------------------------------------
Mcox2Lbal    LIDKTQKQGKKVKKKLFTKVGDDHNSHKLERKWLEDDDDDSDGPGGFGSGPLQPSESFHGGGKVVPSNT

cons         

Hsapiens     ---------------------------------------------------------------------
Fcox2Lbal    ---------------------------------------FE----------------------------
Mcox2Lbal    NNNSANFDYCEGGSVCGVYYEEVAEFTGGLNSVICGVLNFSSACFSFMPIQSFIQFYFANSYSSLCLNW

cons         

Hsapiens     --------------------------------------------------DYGGLIFNSYMLPPL--FL
Fcox2Lbal    ------------------------------------------------GVSDWLISYDSYTVNNVDGVL
Mcox2Lbal    LMVFEEGCVCFITYGSILMSWVSSYAHLLMMFLEELIKIFCKSWPASHMPVTWAIGFDSYLVFFSSLMM

cons                                                               : ::** :     .:

Hsapiens     EPGDLRLLDVDNRVVLPIEAPIRMMITSQDVLHSWAVPTLGLKTDAIPGRLNQTTFTATRPGV-YYGQC
Fcox2Lbal    ESGFRYGQFVDYPMVLPGDSNVEVKVTSADVIHCWTIQGLGVKMDAVPGRVNSAHFSDLRPGFSAWGGC
Mcox2Lbal    PWGFKYGCYVDYPAVLMGDSNIEVKVTSADVVHSWTINGLGMKVDAIPGRINTVQLSGLRPGFSAWGGC

cons           *      **   **  :: :.: :** **:*.*::  **:* **:***:* . ::  ***.  :* *

Hsapiens     SEICGANHSFMPIVLELIPLKIFEMGPVFTL--------
Fcox2Lbal    SEMCGVNHWQMSAEVEVLTVSDFILWILTWVYSDIKDLD
Mcox2Lbal    SEMCGVNHWQMGAEVEVLSRKDFNLWLLT----------

cons         **:**.**  *   :*::. . * :  :
```
